# The Psychological Restorative Effects of Campus Environments on College Students in the Context of the COVID-19 Pandemic: A Case Study at Northwest A&F University, Shaanxi, China

**DOI:** 10.3390/ijerph18168731

**Published:** 2021-08-18

**Authors:** Siyun Sun, Yingyuan Chen, Sen Mu, Bo Jiang, Yiwei Lin, Tian Gao, Ling Qiu

**Affiliations:** College of Landscape Architecture and Arts, Northwest A&F University, Xianyang 712100, China; 2019056003@nwafu.edu.cn (S.S.); cyydy@nwafu.edu.cn (Y.C.); musen@nwafu.edu.cn (S.M.); 18438616006@163.com (B.J.); linyiwei@nwafu.edu.cn (Y.L.)

**Keywords:** campus environment, psychological restoration, landscape design, time, suitability of using time

## Abstract

During the COVID-19 outbreak, college students experienced different periods of isolation on campus, which has had an impact on students’ mental health. Based on ART theory, this study randomly selected students at Northwest A&F University, Shaanxi, China and distributed questionnaires in order to evaluate the psychological recovery effect of campus environment during the epidemic. The results showed that: (1) There were significant differences in the psychological restoration of four types of campus environments. Blue space had the greatest effect, followed by green space and sports grounds, while grey space had the least. (2) Time of stay had a very significant impact on psychological restoration. Longer time of exposure is not necessarily correlated with a better recovery experience. (3) In the different campus environments, extent is easier to be perceived followed by fascination and compatibility, and the weakest is being away. At the time of stay level, no significant difference was found in the perception of compatibility. Time of stay was negatively correlated with fascination and compatibility. These findings can provide theoretical and practical bases for campus environmental planning and construction following the COVID-19 epidemic.

## 1. Introduction

The novel coronavirus (COVID-19) is a public health pandemic that has affected the physical and mental health of individuals [1]. Studies have shown that the physical damage may be recovered in a short time, but the psychological impact is longer lasting [2]. With the development and spreading of COVID-19, daily life has been altered for people to a certain extent, resulting in different levels of adverse mental health conditions, such as depression, anxiety, fear, and insomnia [3,4,5,6]. Since the COVID-19 outbreak, China has taken the most comprehensive, rigorous, and comprehensive prevention and control measures [7]. To prevent the COVID-19 outbreak from spreading to schools, all universities across the country were asked to start online classes and postpone students’ return to campus. As the pandemic has stabilized, the college students, who have been quarantined at home for over 4 months, were asked to return to campus in batches according to the requirement of the Ministry of Education of the People’s Republic of China, spanning a time frame from April to August of 2020. Specifically, teaching staff and graduate students comprised the first group who returned to campus, followed by the students with subsequent research assignments or special circumstances (volunteers for epidemic prevention and control, etc.). The rest of the undergraduate students were the last to return to campus because they travel from all over China, which could be a great potential risk for viral transmission across the country. The university has warned the students to refrain from going outdoors as much as possible and to keep safe. In conclusion, students had more or less been influenced from a psychological perspective by COVID-19 [8]. Therefore, the understanding of students’ psychological conditions is of great importance for their overall health and daily life during the pandemic.

More and more studies have shown that the exposure to a natural environment is expected to lead to psychological wellbeing, and improvements in mood, pleasure, and even better health [9,10,11,12,13]. Since the 1980s, Kaplan conducted theoretical and empirical studies centering on the healing environment, and proposed the attention restoration theory (ART). ART assumes that the capacity to direct attention to something diminishes with continuous use. As the capacity to direct attention and focus diminishes, a person may commit errors on tasks, become irritable, and otherwise show signs of attentional fatigue. Restoration actually is the process of recharging depleted cognitive function and capability, which are negatively affected by prolonged directed activities or exposure to stress that produces mental fatigue [14]. In a restorative environment, the individual will effectively recover, experience extensive repair, restore the ability to direct attention, and improve the ability to reflect on what is important [14,15,16]. ART proposes that the restorative environment should fully include four dimensions: being away, extent, fascination, and compatibility [16]. According to the theory of ART, Hartig designed the Perceived Restorativeness Scale (PRS) that is used to measure the restorative potential of an environment based on the four components [17]. The PRS has been further developed by many researchers and widely used in a variety of environmental restoration studies but seldom in college environments [18,19,20].

Campus space, as a prominent part of green spaces, is of great importance for students’ physical and mental recovery. Some studies have shown that greening of the schoolyard significantly improves students’ physiological well-being and reduces physiological stress [21]. Likewise, exposure to natural environments can also have a positive impact on the academic performance due to enhancing individual aesthetic preference and mental restoration [22,23,24]. However, simply understanding the restorative values of natural environments is not enough to provide an essential guideline for restorative environment design in practice [25]. To address this concern, in recent years, some studies have evaluated the environment in different categories. Although site classification evaluation has been extensively studied, the natural environment, including blue spaces and green spaces, has always been emphasized for mental restoration [26,27]. Nevertheless, sports grounds are frequent places for college students who engage in various recreational activities as part of their daily lives [28]. Physical exercise has a positive impact on people’s physical and mental health [29,30]. Therefore, it should also be taken into account in order to identify students’ perceptions of mental restoration within the sports grounds in this study.

Note that several known and unknown factors may also influence people’s perceptions and preferences such as demographic information, characteristics of site, and season of visit, etc. [31]. Some studies have shown that people’s perceptions and preferences can be influenced by the change in characteristics of an environment caused by a change in season [32,33]. However, few studies have considered the effect of the environment on people’s mental restoration in the same season across different times. Many studies examined the changes in psychological responses to the different types of environments only in a short period of time, and prolonged timeframes have seldom been used [34]. Actually, time of stay has played an important role in the mental recovery process [35,36,37], especially for the college students who were confined to stay on campus for 4 months during the harshest period of the pandemic. This means that these students were constantly exposed to the same college environment across an extended amount of time, and their health status should be of particular concern. Moreover, compared with other college students who returned to campus in batches, the influence of time of stay of restorative college environments should be examined.

Therefore, the overarching aim of this study was to elucidate the influence of campus environments on college students’ psychological restoration during the pandemic period. Investigating the following aspects specifically:

1. The difference in psychological restoration among the different types of campus environment.
2. The relationships between time of stay (time of return to campus or stay on the campus) and psychological restoration.
3. The relationships between the four dimensions (being away, extent, fascination, and compatibility) and psychological restoration based on PRS.

## 2. Materials and Methods

### 2.1. Study Area

**Figure 1:**
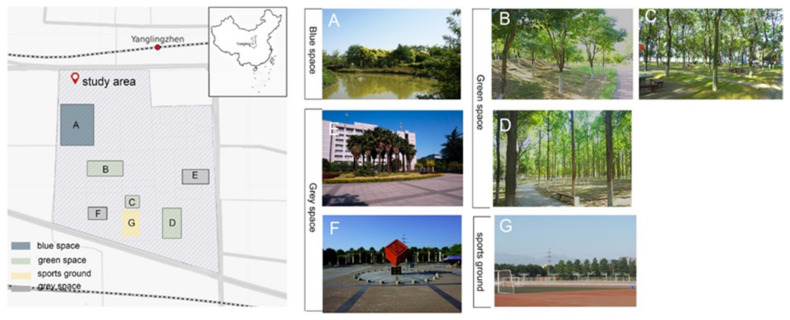
Study area and photographs of the study sites at Northwest A&F University, Shaanxi, China ((**A**) represents blue space, (**B**–**D**) represent green space, (**E**,**F**) represents grey space, and (**G**) represents sports ground).

Northwest A&F University in Shaanxi, China was selected as the study area, and is geographically located in the center of China, covering a total area of 1.24 km^2^. According to the characteristics of the public open/green spaces in campus, they were divided into four categories: blue space, green space, gray space, and sports ground (Figure 1). Although sports grounds could be considered as open spaces, for the particular purpose of this study, such grounds were classified in a separate category.

### 2.2. Data Collection and Questionnaire

Students who stayed in and used the study area were randomly questioned and informed the survey objectives, and those who were willing to participate were considered subjects. Those willing to participate were then given the questionnaire and invited to fill it in during their stay in the area. All of the investigations were carried out on sunny days with no wind from a time period spanning May to August of 2020. Each investigation of the selected sites followed the same procedure.

The questionnaire consisted of three parts. The first part contained the personal information of the participants including gender, homeplace, and time of return. According to the students’ actual time of return, they were divided into five groups: back to school for a week (T0), back to school for a month (T1), back to school for 2 months (T2), back to school for 3 months (T3), and those who had stayed at school for over 4 months (T4). The second part included a question regarding the preference for the selected environments and reasoning for the preference. The third part of the questionnaire evaluated the psychological restoration of the participants using the Perceived Restorativeness Scale (PRS). The scale included 22 items: five items for Being away, five items for Fascination, six items for Compatibility, and five items for Extent. Each item was accurately translated in Chinese and assessed by a 7-point Likert scale ranging from 1 = “do not agree at all” to 7 = “agree very much”. Each participant was asked to perceive only one type of the selected environments. The score for each environment will be counted as an individual evaluation of the restorative effects of the environment.

### 2.3. Data Analysis

All statistical analyses were carried out using the SPSS 24 software (IBM, Armonk, NY, USA). Before the statistical analyses, the values of the Perceived Restorativeness Scale (PRS) were calculated. The mean values of the items of each restorative component were used to identify the restorative effect of each particular component, and the mean values of all the 22 items were used to identify the overall psychological restoration of each participant. Independent sample T test was used to test whether population information had an effect on recovery score. A one-way ANOVA with LSD tests was conducted to analyze the difference in psychological restoration among the different types of campus environments. To identify the relationships between time of stay (time of return to campus or stay on the campus) and psychological restoration, a one-way ANOVA with LSD tests was conducted. To explore the relationships between the four dimensions (being away, extent, fascination, and compatibility) and psychological restoration, an analysis of ANOVA was used, using the four types of campus environment and time of stay as the dependent variable respectively, and the four dimensions as the independent variables. Finally, the Spearman’s Rho correlation analyses between time of stay and four dimensions (being away, extent, fascination, and compatibility) were conducted.

## 3. Results

A total of 819 questionnaires were finally collected, including 232 from the participants in T0, 237 in T1, 195 in T2, 116 in T3, and 36 in T4. The sample size of the T4 group was the smallest because the students who had remained on campus throughout the pandemic were limited. Independent sample T test showed that gender and hometown had no significant effect on recovery values (Table 1).

### 3.1. The Different Psychological Restorations among the Different Types of Campus Environment

A one-way ANOVA with a post hoc test revealed that the participants significantly differed in the overall restoration experienced among the four types of campus environments (F = 15.18, *p* = 0.00). Blue space was perceived as the most restorative environment, followed by sports ground and green space, and grey space was the least (Table 2).

### 3.2. The Relationships between Time of Stay (Time of Return to Campus or Stay on the Campus) and Psychological Restoration

#### 3.2.1. The Impact of Time of Stay on the Recovery of the Entire Campus Environment

A one-way ANOVA with a post hoc test showed that the participants significantly differed in the overall restoration experienced among the five timings (F = 4, *p* = 0.01). T4 participants were perceived as experiencing the most restorative while the T2 and T3 groups perceived restoration less. The restoration across the selected campus environments decreased over time and increased after the T3 group (Table 3).

#### 3.2.2. The Impact of Time of Stay on Mental Restoration Experienced within the Four Types of Campus Environment

A one-way ANOVA showed that the restoration experienced in the four environments at each time node significantly differed except for T4. Blue space had the highest restorative effect in the T0 group. The T4 group experienced the highest restorative effect within the green space. This indicated that the students who had just returned to school considered the blue space to be the most restorative, but the students who continually remained at school during the pandemic believed that the green space held the most restorative effect (Table 4).

### 3.3. The Relationships between the Four Characteristics (Being Away, Extent, Fascination, Compatibility) and Psychological Restoration Based on PRS

#### 3.3.1. The Four Dimensions among the Different Types of Campus Environment

There were significant differences in the fascination, compatibility, and being away dimensions in the selected four types of campus environments, but none were found for the dimension of extent (*p* > 0.05). Overall, across four types of selected environments, extent was more perceived, while compatibility and being away were less perceived. The results also indicated that blue space scored the highest on the dimension of extent, and sports grounds scored the highest on the dimensions of compatibility and being away (Table 5).

#### 3.3.2. The Impact of Time of Stay on Four Dimensions

According to the one-way ANOVA results, besides the dimension of compatibility, time of stay significantly influenced other dimensions (Table 6). Correlation analysis showed that the extent and fascination dimensions were significantly negatively correlated with time of stay (Table 7). This indicated that the longer a student stayed in the same environment, the less he or she perceived the extent and fascination features from the environment.

## 4. Discussion 

### 4.1. Reconsidering the Restorative Effects of Campus Environments

There are significant differences in mental restoration within the four types of campus environment. Blue space was considered as the best, followed by sports ground and green space, and gray space was the least. First, the results revealed that blue space has the best restorative potential among the four types of environments. This is in line with a previous study, which showed that water, regardless of the type (such as a river, lake or fountain) is considered to be one of the most important landscape elements [38]. Water is also typically seen as a highly valuable factor in the assessment of the likelihood of restoration [39]. Human beings naturally have a positive response to aquatic elements for seeking tranquility and healing [40]. The same is true for college students. In addition to the blue space, the sports grounds also had a positive impact on students’ mental restoration. This could be explained by the improvement in cognitive functioning, which is not necessarily limited to nature [16]. Many studies have shown that physical activity may also improve mood and mental health [41,42]. The sports grounds not only provide an environment for students to exercise but also serve to improve students’ mental health to a certain extent. The mental restorative effect is greater than that of grey space, which is consistent with the results of previous studies. Some studies have shown that plants are an important element in improving people’s landscape preferences and mental restoration [15]. Green spaces with more trees or higher vegetation density were more preferred and perceived as more restorative [43,44].

It is worth noting that in recent years, the possible synergies between different health benefits have attracted increasing interest across disciplines. A large body of evidence shows that physical activity in natural environments is more positively associated with improved mood and emotional well-being [45,46,47]. In the context of the epidemic, the campus restorative landscape design is particularly important. Previous landscape design has focused on adding green space but this is not the only way to increase the potential for environmental restoration [12]. Our study showed that sports venues also play an important role in the restorative experience. Future landscape design should abandon the idea of blindly increasing green space, and consider providing exercise places or promote the use of green space as an opportunity to encourage people’s activities, which could effectively improve the overall ability of environmental restoration.

### 4.2. The Impact of Time of Stay on Mental Restoration

In this study, the results indicated that the psychological restoration of college students in the campus environment across 4 months (t0–t3) showed a downward trend with the change of time of stay. This indicated that the longer time of exposure is not necessarily correlated with a better recovery experience, especially for the students who were returned to campus in batches (T0–T3). Visiting natural settings that are far from home might promote perceived relaxation via the sense of being away from everyday life, as suggested by the attention restoration theory [14]. During the COVID-19 pandemic, social distancing measures remain as the available option to governments to help slow the spread and ideally control the pandemic [48]. At the suggestion of schools, most students choose to participate in activities on campus rather than go out, therefore the constant exposure to the campus environment in their daily life can have a significant impact on their mental health. However, our results showed that during this period, the mental restoration of the respondents brought by the environment decreased. That is probably because the perceived relaxation would decrease over time due to the vanishment of the sense of being away. These students have become more and more familiar with the campus environment. Because of this, in this case, it is worth continual discussion concerning the use of landscape design to optimize the campus environment and minimize the psychological harm of isolation to students.

In addition, it is worth noting that students who stayed in school for over 4 months (T4) had higher restorative experiences than at other time points. There are two possible reasons for this. Firstly, the campus environment changes across seasons. Previous studies have shown that season may influence the landscape preference. Seasonal changes in tree foliage enhance the perceived restorative quality of campus environments [49]. With the change of seasons, the biological characteristics of plants will change the appearance, color, shape, density, biodiversity, and other ecological characteristics of plant communities, thus affecting visual perception and psychological responses [50,51]. Although our study was conducted over a single season, it is important to note that there are more plants across the campus, and thus the appearance of plants may change, even across short time frames, which provides students with various visiting experiences to some extent. That is why the T4 group viewed quite differently the spaces having the most restorative effect (Green Space > Sports Ground > Grey Space > Blue Space). There are the most abundant plants in the green space in the study area. A second possibility is that students’ adaptation to the campus environment increases the restorative experience. The core definition of the current research on the emotional relationship between people and places is primarily place attachments [52,53,54]. Place attachment is a special dependent relationship between some places and people in daily life. For example, people often regard their residential environment as their greatest place of attachment, so if a person resides within an environment similar to where they live, it will cause them to recall the most beautiful experiences and stimulate a positive emotional experience [55]. As students stayed in school for a longer time (T4), the relationship between campus environment and the students gradually changed and the restorative experiences also changed correspondingly. Therefore, the T4 participants could regard the sports ground and grey space as their place of attachment for outdoor recreational activity in their school life. Given that, our study tentatively suggests that appropriately extending stays within nature, which can assist individuals in perceiving the seasonal changes under the circumstances, may be good for students’ mental restoration.

### 4.3. The Relationships between Four Dimensions (Being Away, Extent, Fascination, Compatibility) and Mental Restoration

Fascination is considered attractive as long as information in the environment does not require effort to gain an individual’s attention [16]. Blue space is more restorative for students who have just returned to school and performs well in the dimension of fascination. These findings are consistent with previous studies that claimed that water is an important element for increasing the attractiveness and restorative potential of an environment [56,57,58]. This may indicate that fascination plays an important role in mental restoration (within four months). Therefore, for certain places we occasionally visit, such as scenic venues, nature reserves can effectively improve environmental restoration by improving the environmental fascination of individuals. In addition, water is typically seen as an attractive visual element [57,59]. The design of water features can improve the restorative potential of the environment, specifically the level of the dimension of fascination.

Being away implies a setting that is physically or conceptually different from one’s everyday environment [16]. Across four types of environments, the dimension of being away was less perceived. This could be explained by the fact that the selected sites in this study were all places which the respondents were acquainted with in their daily life. However, the dimension of being away was perceived higher in the sports ground environment. Kaplan (1995) emphasized that the sense of being away can also be realized through psychological adjustment, such as changing the content of thinking, sitting, meditation, and so on. During the pandemic, since the respondents who had returned to school or remained at the school were not allowed to leave campus casually, they had to search for some attractive places on the campus that were different from their daily life for mental restoration. They could have a feeling of being away in sports grounds when doing exercises. This also suggests that future campus environmental design should increase the interactions between environment and students, and encourage students to participate in various activities.

Compatibility refers to promoting physical activity or leisurely pursuits, such as photography or socializing [60,61,62]. The sports ground performed well in the dimension of compatibility. This can be explained by realizing that the sports ground satisfies compatible characteristics of the restorative environment. Many respondents considered sports ground as a place wherein individuals could exercise, run, and walk. This indicates that the compatibility in the campus environment is largely affected by students’ own activities. The restoration of the sports ground may stem from the students’ own activities to achieve mental restoration, rather than from the environment itself. A recent study suggested that the link between environment and good mood can be made through capacity-building such as social cohesion and physical activity [62]. It can be seen that mental restoration may arise from the environment, while at the same time it may depend upon people’s spontaneous activities. It is worth noting that all the other dimensions were influenced by the change in time of stay except the compatibility dimension. This finding explains why the recovery changes over the time of stay. Since there was no significant difference in mental restoration across the compatibility dimension, we can infer that the dimension of compatibility played a more stable role in the construction of restorative environments during the 4 months under the influence of COVID-19. Under the influence of time of stay, fully meeting people’s activity needs was the most effective way for restorative environment construction. This is particularly important for landscape design in the context of public health events.

Extent implies a setting sufficiently rich and coherent that it can engage the mind and promote exploration [16]. A study utilizing the social media platform Twitter revealed that the most common thing people mentioned was attractiveness or compatibility. Fewer than 5% of tweets mentioned leaving, and none mentioned an extent [63]. An explanation may be that the student paid little attention to extent or that it may have been less perceived [64,65]. Extent showed no significant differences in the four categories of environments. This finding might explain how it may not play a dominating role in improving the restoration experience of the environment. When using ART theory for research, few studies have assessed the relative importance of restoration across the four features. In all studies, there is no consensus as to which dimension is the most important [18,47,62,64,66]. Our study showed that the importance of the four dimensions is different under different circumstances. There is no significant difference in the extent dimension among different environments. In this case, the improvement of other dimensions can attract more attention and be greater perceived by people than extent.

## 5. Limitations and Future Work

The study was based on students’ self-reported changes in psychological recovery, so participants may have underestimated or overestimated responses. It would be more objective to add physiological changes to the test. In the future, physiological index tests should be added to make the results more comprehensive. Moreover, only students were selected as the subjects of the study. Although College students can in some way replace the views of the overall population [67], note that age, childhood background, and education of the study subjects should be taken into account. Finally, the impact of property & facilities management on environmental psychological recovery should be considered, which was neglected in the current and previous studies on environmental psychological recovery.

## 6. Conclusions

This study applied a questionnaire as a tool to explore the effects of different campus environments and changes in times of stay on mental restoration. Blue space and sports ground had more psychological restorative effects on the students. Time of stay on the campus significantly affected the mental restoration. However, the longer time of exposure is not necessarily correlated with a better recovery experience. Psychological restoration was influenced by the combination of different types of environments and time of stay. In the four types of different campus environments, the extent dimension was more easily perceived followed by fascination and compatibility, while the weakest dimension was being away. The extent dimension had no significant difference among the four types of environments and the compatibility was little affected by time of stay. With time of stay increased, fascination and compatibility were negatively correlated.

Therefore, one should pay attention to the impact of sports site design and time of stay on the restorative environment in the future. For example, incorporating sports, water environments, and compatibility into the design could effectively increase the positive impact of the daily environment on human psychology.

## Figures and Tables

**Table 1:** *T*-test on population information.

Category		*N*	Mean Value	S.D.	S.E.	95% C.I.	*p*
Gender	Man	350	4.27	0.91	0.05	−0.09–0.16	0.75
	Female	469	4.23	0.92	0.04		
Hometown	Urban	441	4.19	0.94	0.04	−0.24–0.01	0.30
	Countryside	378	4.31	0.89	0.05		

**Table 2:** ANOVA with a post hoc test on recovery values among the four study sites.

Type of Space	N	Mean Value	S.D.	S.E.	95% C.I.	Rank
Blue space	103.00	4.52 ^a^	0.92	0.09	4.34–4.70	1
Sports ground	189.00	4.51 ^a^	0.78	0.06	4.39–4.62	2
Green space	354.00	4.17 ^b^	0.95	0.05	4.07–4.26	3
Grey space	173.00	3.97 ^b^	0.88	0.07	3.83–4.10	4

Significant difference at the 0.05 level is shown by different letters a and b.

**Table 3:** ANOVA results of mental restoration of the students at different times of stay in school.

	N	Mean Value	S.D.	S.E.	95% C.I.
T0	232.00	4.34 ^a^	0.93	0.06	4.22–4.46
T1	237.00	4.31 ^a^	0.94	0.06	4.19–4.43
T2	195.00	4.12 ^b^	0.86	0.06	3.98–4.23
T3	119.00	4.07 ^b^	0.90	0.08	3.91–4.23
T4	36.00	4.58 ^a^	0.85	0.14	4.29–4.87

Significant difference at the 0.05 level is shown by different letters a and b.

**Table 4:** ANOVA results of recovery under different environments at five time points.

PRS	Blue Space	Green Space	Grey Space	Sports Ground	F	*p*
T0	4.64	4.35	3.98	4.53	5.26	0.00
T1	4.57	4.25	4.05	4.56	3.47	0.02
T2	4.32	3.92	3.97	4.50	5.92	0.00
T3	4.50	3.98	3.67	4.29	3.70	0.01
T4	4.19	4.79	4.32	4.55	0.77	0.52

**Table 5:** ANOVA results among the four dimensions comparison of different environments.

PRS	Blue Space	Sports Ground	Green Space	Grey Space	F	*p*	Total of Mean	Rank
Extent	5.73 ^a^	5.76 ^a^	5.61 ^a^	5.49 ^a^	2.20	0.09	5.65	1
Fascination	4.43 ^a^	4.05 ^b^	3.85 ^b^	3.63 ^c^	9.10	0.00 **	3.99	2
Compatibility	4.08 ^a^	4.34 ^a^	3.69 ^b^	3.52 ^b^	13.78	0.00 **	3.90	3
Being away	3.85 ^a^	3.87 ^a^	3.51 ^b^	3.22 ^c^	8.78	0.00 **	3.61	4

Significant difference at the 0.05 level is shown by different letters a, b and c. ** The significance of difference is at the 0.01 level.

**Table 6:** ANOVA results across the four dimensions compared to different lengths of time.

PRS	t0	t1	t2	t3	t4	F	*p*
Extent	5.65 ^b^	5.80 ^b^	5.43 ^b^	5.56 ^b^	5.80 ^a^	3.72	0.00
Fascination	4.12 ^a^	4.00 ^c^	3.70 ^b^	3.71 ^b^	4.08 ^a^	3.81	0.00
Compatibility	4.02 ^a^	3.83 ^a^	3.74 ^a^	3.67 ^a^	4.06 ^a^	1.90	0.11
Being away	3.57 ^b^	3.58 ^b^	3.56 ^b^	3.34 ^b^	4.36 ^a^	3.93	0.00

Significant difference at the 0.05 level is shown by different letters a, b and c.

**Table 7:** Correlations among the four dimensions and the five timings restoration experienced.

	*p*	Correlation Coefficient
Extent	0.00	−0.105 **
Fascination	0.01	−0.008 *
Compatibility	0.39	−0.04
Being away	0.44	0.027

* The significance of difference is at the 0.05 level. ** The significance of difference is at the 0.01 level.
